# A review on biodiesel production from microalgae: Influencing parameters and recent advanced technologies

**DOI:** 10.3389/fmicb.2022.970028

**Published:** 2022-07-29

**Authors:** Shiqiu Zhang, Lijie Zhang, Geng Xu, Fei Li, Xiaokang Li

**Affiliations:** ^1^College of Chemistry, Chemical Engineering and Materials Science, Shandong Normal University, Jinan, China; ^2^School of Environmental and Material Engineering, Yantai University, Yantai, China; ^3^School of Municipal and Environmental Engineering, Shandong Jianzhu University, Jinan, China; ^4^School of Geography and Environment, Shandong Normal University, Jinan, China

**Keywords:** microalgae cultivation, microalgae harvesting, lipid accumulation, lipid extraction, biodiesel

## Abstract

Microalgae are the important part of carbon cycle in the nature, and they could utilize the carbon resource in water and soil efficiently. The abilities of microalgae to mitigate CO_2_ emission and produce oil with a high productivity have been proven. Hence, this third-generation biodiesel should be popularized. This review firstly introduce the basic characteristics and application fields of microalgae. Then, the influencing parameters and recent advanced technologies for the microalgae biodiesel production have been discussed. In influencing parameters for biodiesel production section, the factors of microalgae cultivation, lipid accumulation, microalgae harvesting, and lipid extraction have been summarized. In recent advanced technologies for biodiesel production section, the microalgae cultivation systems, lipid induction technologies, microalgae harvesting technologies, and lipid extraction technologies have been reviewed. This review aims to provide useful information to help future development of efficient and commercially viable technology for microalgae-based biodiesel production.

## Introduction

The consumption of fossil energy has been on the rise following the rapid development of the society, leading to reduced fossil energy resource reserves and causing severe deterioration of the natural environment (Zhang S. et al., 2020). International efforts to achieve carbon neutrality urgently necessitate the development of new clean energy sources to replace fossil energy (Hou et al., 2021). Biodiesel, produced by renewable resources such as vegetable oil or animal fat, is a new clean and alternative to diesel fuel. This renewable resource primarily consists of esters formed by methanol or ethanol and long-chain saturated and unsaturated fatty acids such as palmitic acid, stearic acid, oleic acid, and linoleic acid (šalić et al., 2020). Therefore, the research and development of biodiesel has attracted the attention of scholars worldwide.

**FIGURE 1:**
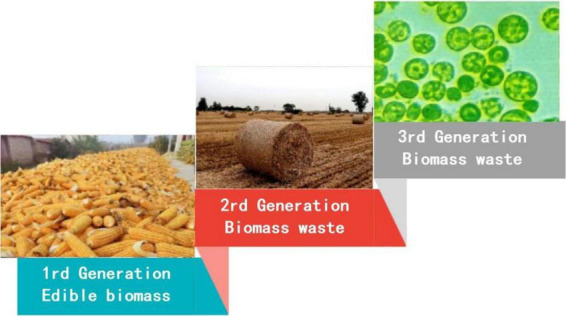
Evolution of biodiesel.

The development of biodiesel can be divided into three stages (Figure 1). First-generation biodiesel was prepared using edible crops such as corn, soybean, and sugarcane as raw materials. These materials exhibited low yield and consumed copious amounts of water and land resources, thus straining the resources required for societal sustenance (Li and Guo, 2017). Second-generation biodiesel was mainly produced using lignocellulose, such as straw, hay, and other non-foods, as raw material. Although the production of this generation of biodiesel addressed the limitations of production associated with first-generation biodiesel, it was costly and provided low calorific value owing to the features and collection cost of lignocellulose. Third-generation biodiesel is produced from microalgae, which produce lipid through carbon fixation during photosynthesis and have a yield of lipid up to 70%. In addition, microalgae live in water, and it does not occupy farmland. They also exhibit a short growth cycle and high yield, and their consumption does not threaten human food supply. These advantages of microalgae make it optimal alternative raw material for biodiesel production.

**FIGURE 2:**
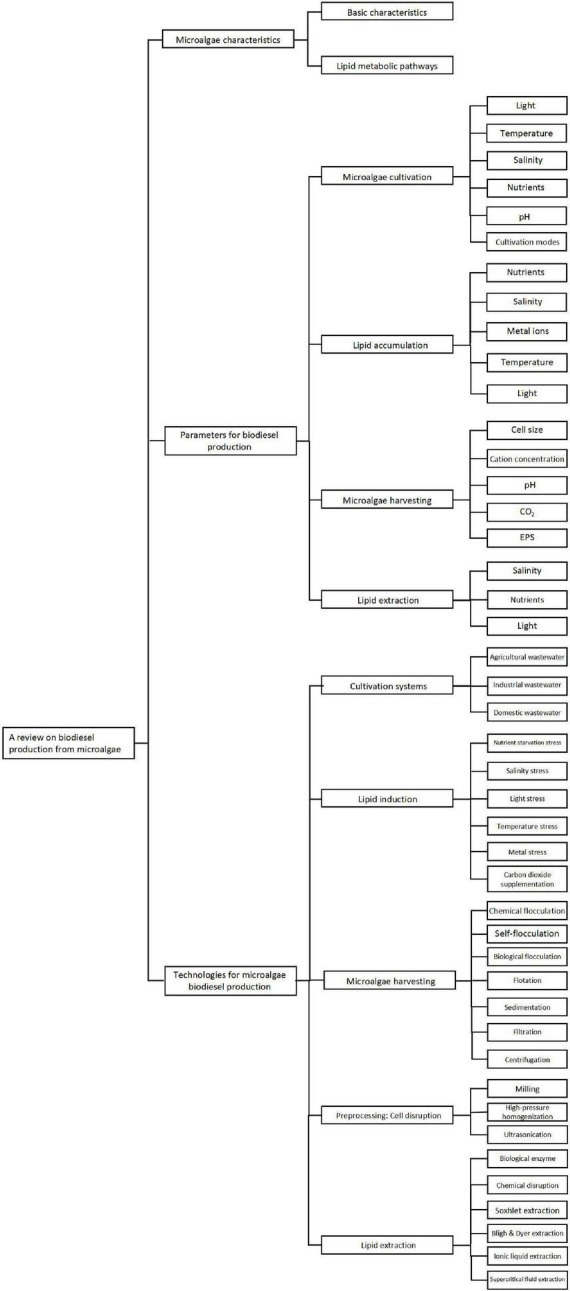
Schematic diagram of main content in this review.

Biodiesel production from microalgae entails reusing and integrating carbon resources from the natural environment, which can contribute to achieving the long-term goal of replacing petroleum use and reducing environmental pollution, thereby contributing significantly to the global carbon neutrality endeavor. Hence, we aimed to review the key technologies of biodiesel production from microalgae in this work, and the schematic diagram of main content is shown in Figure 2.

## Characteristics of microalgae

### Basic characteristics

**FIGURE 3:**
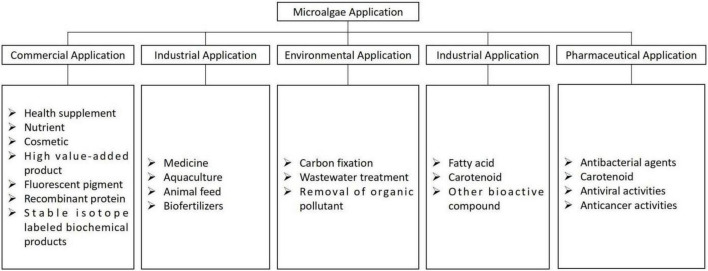
Applications of microalgae.

Microalgae is microscopic and unicellular microorganisms that have been living on the earth through prolonged geological ages. The photosynthetic mechanism of microalgae is similar to that of plants. Owing to its simple structure, it can efficiently obtain water, carbon dioxide, and other nutrients when submerged in water, thus allowing them to convert solar energy into biomass resources. Generally, microalgae are categorized as freshwater and marine microalgae based on their living environment, among which freshwater microalgae are widely distributed in nature, including rivers, lakes, reservoirs, creeks, ponds, marshes, and soils (Singh et al., 2020). Microalgae have been used in numerous fields (Figure 3), such as food, cosmetics, aquaculture, environmental testing, wastewater treatment, and energy.

**FIGURE 4:**
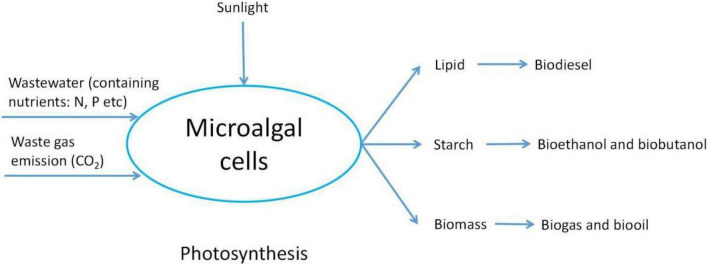
Schematic of biomass energy production from microalgae.

As a bioenergy source, microalgae exhibit high photosynthetic efficiency and high yields of biomass and lipid with few environmental restrictions. Microalgae can live on non-arable land, such as beaches, saline and alkali soils, and deserts, and it could also grow in wastewater and seawater (Zada et al., 2021). In addition, microalgae exhibit remarkable performance in terms of carbon fixation (Zeng et al., 2021). At a growth rate of 25 g/d, microalgae can fix 12 tons of CO_2_ per acre per year. The differences in biomass yield, lipid/fat yield, land use, and biodiesel yield among various crops are presented in Table 1. It indicates that microalgae clearly surpass other crops in all aspects. Therefore, microalgae are the most promising alternatives to replace fossil fuels. The lipid in microalgae or the whole microalgae cell can be utilized to produce gaseous fuels such as hydrogen and biogas, or liquid fuels such as ethanol and liquid hydrocarbon fuels (Sanghamitra et al., 2020). Therefore, the development of microalgae biomass energy at this stage includes biodiesel production from microalgae lipid by transesterification (Vignesh et al., 2021), methane production by anaerobic digestion (Qi et al., 2017), and production of hydrocarbon or crude oil-like substances by gasification and pyrolysis (Su et al., 2022; Figure 4).

**TABLE 1:** Comparison of microalgae with other feedstocks.

Plant name	Lipid content (%)	Lipid yield (L/ha⋅year)	Land use (m^2^ year/kg ⋅biodiesel)	Biodiesel yield (kg/ha⋅year)
Corn	44	172	66	152
Cannabis	33	363	31	321
Soybeans	18	636	18	562
Jatropha	28	741	15	656
Camelina	42	915	12	809
Canola	41	974	12	862
Sunflower	40	1,070	11	946
Castor	48	1,307	9	1,156
Palm	36	5,366	2	4,747
Microalgae (low lipid content)	30	58,700	0.2	51,927
Microalgae (medium lipid content)	50	97,800	0.1	86,515
Microalgae (high lipid content)	70	126,900	0.1	121,104

Biodiesel production from lipid in microalgae through transesterification is characterized by the advantages of high growth rate and lipid yield, tolerance to environmental stress, and low competition for land (Ebhodaghe et al., 2022). Compared with that of traditional diesel, the performance of biodiesel is superior in several aspects: (1) Biodiesel is extracted from vegetable and animal oils and fats, and is therefore renewable. (2) It contains relatively low sulfur and nitrogen content, thereby reducing SO_2_ and nitrogen oxide emissions. (3) It also has a high flash point and high combustion performance and efficiency, is not prone to explosion, and is safe to use. (4) It also exhibits adequate performance at low temperature and lubrication. These advantages accord it remarkable application prospects in various fields.

### Lipid metabolic pathways in microalgae

**FIGURE 5:**
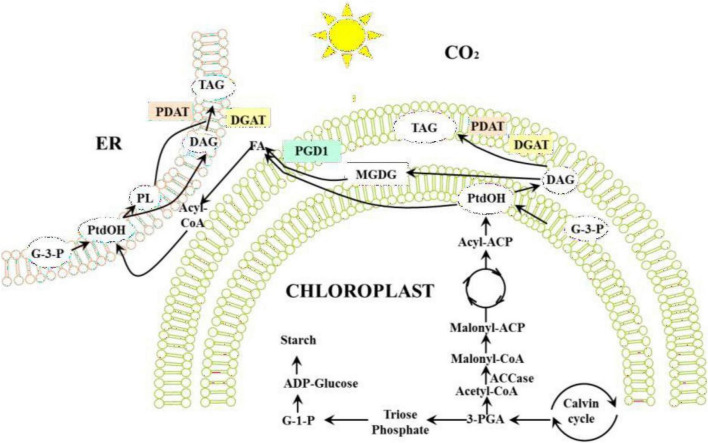
Triacylglyceride (TAG) synthetic pathway in microalgae cell (Zienkiewicz et al., 2016).

In microalgae cell, the synthesis of fatty acid usually occurs in the plastids, and the synthesis of triacylglyceride (TAG) would occur in both of chloroplasts and endoplasmic reticulum (Figure 5). In contrast to phospholipids in biological membranes, TAG does not have structural roles in cells and are used to store energy and carbon. In microalgae cells, two main TAG synthesis pathways exist: the Kennedy pathway and the Monoacylglycerol pathway. In these two pathways, TAG is synthesized through esterification between acetyl-CoA and hydroxyl groups on glycerol. The biosynthesis of fatty acids begins with the carboxylation of acetyl-CoA and acetyl-CoA carboxylase (ACCase)-mediated catalysis to form malonyl-CoA. ACCase is a critical control factor in fatty acid synthesis. In microalgae cells, malonyl-CoA is first transferred to the acyl carrier protein (ACP), and then undergoes a series of acyl chain-elongation reactions. Finally, the C16 or C18 products are catalytically synthesized by multiple subunits of fatty acid synthases. The elongation of fatty acids is terminated by two types of enzymes. First, the acyl groups in the chloroplast acyltransferase are removed by ACP, and the newly synthesized fatty acid is directly transferred from ACP to glycerol-3-phosphate (G-3-P). Second, acyl-ACP thioesterase hydrolyzes acyl-ACP and releases free fatty acids. Free fatty acids are transferred out of the chloroplast to generate glycerides.

In the Kennedy pathway, the first acyl group is esterified to glycerol-3-phosphate (Reddy et al., 2010). Phospholipid synthesis occurs in the second reaction. Before conversion to the TAG, phospholipid is dephosphorylated to diacylglycerol (DAG) by phosphatase enzyme. The Monoacylglycerol pathway starts with 2-monoacylglycerol (sn2-MAG), which is converted to DAG by monoacylglycerol 2 acyltransferase (MGAT). The synthesized DAG is converted to TAG through catalysis by acyl-CoA: DAG acyltransferase, and this is an important step in TAG biosynthesis.

**FIGURE 6:**
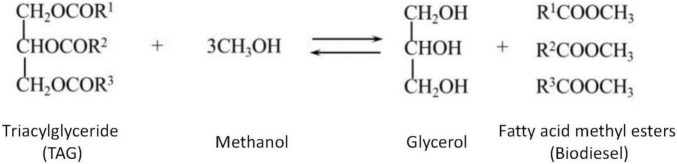
Triacylglyceride (TAG) is used to synthesize biodiesel through transesterification.

In another potential pathway for TAG synthesis catalyzed by phospholipid: diacylglycerol acyltransferase (PDAT), phospholipid or galactolipid acts as the acyl donor (Dahlqvist et al., 2000). Some plants have been shown to have PDAT activity, however its contribution in TAG synthesis differs between different plants. The PDAT encoding gene has been identified in microalga, proving that an acetyl-CoA-independent mechanism for TAG synthesis exists in microalga. Fatty acid methyl esters (biodiesel) are produced through TAG transesterification (Figure 6).

## Parameters for biodiesel production

**FIGURE 7:**
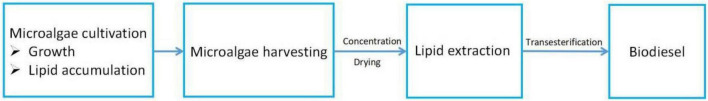
Production process of microalgal biodiesel.

Researches on biodiesel produced from microalgae mainly focus on the production process, which primarily encompasses the upstream process involving microalgae cultivation (growth and lipid accumulation) and the downstream process including microalgae harvesting and lipid extraction (Figure 7). The technology of converting lipid to biodiesel is well established and requires no in-depth discussion in this study. The precursor material of biodiesel-the lipid-is currently research focus. The fatty acid components in microalgal lipid play a crucial role in the quality of biodiesel, which in turn affects the combustion efficiency and heating power of the engine. Under certain stresses, microalgae produce lipids primarily consisting of neutral fatty acids with a low degree of saturation (no more than two unsaturated bonds in most cases), thus confirming the applicability of biodiesel produced from microalgae and its potential to partially replace fossil fuels (Song et al., 2022). Biodiesel exhibits remarkable portability, ready availability, renewability, high combustion efficiency, low sulfur and aromatic content, high cetane number, and adequate biodegradability. It also exhibits more optimized performance in terms of safety and energy balance ratio than regular diesel fuels. The factors affecting each stage of the microalgal biodiesel production process are detailed below (Chhandama et al., 2021).

### Microalgae cultivation

Microalgae cultivation can directly affect the yield of lipid. Various factors such as light, carbon sources, nutrients (e.g., nitrates, phosphates, and carbohydrates), and trace elements (e.g., Mn, Co, Zn, and Mo) influence the growth of microalgae (Aziz et al., 2020). Additional influencing factors include temperature, pH, salinity, O_2_ removal, and CO_2_ uptake. The comparison of the parameters on microalgae cultivation is in Table 2.

**TABLE 2:** Comparison of the mainly parameters and technologies for biodiesel production.

Main parameters	Factors	Microalgae type		Lipid content	Biomass growth/Productivity	
Microalgae cultivation	Light	*Chlorella* sp.	2700 lx,	19.44%	1.05/d	Monika et al., 2015
		*Chlorella vulgaris*	520 mmol/m^2^ s	22.2%	0.36/d	Maria et al., 2019
	Temperature	*Chlorella vulgaris*	25 °C	14.7%	0.14/d	Attilio et al., 2009
		*Chaetoceros* sp.	25 °C	16.8%	0.87/d	Susan et al., 2002
	pH	*Chlorella* sp.	8	23%	0.1995 g/L	Monika et al., 2015
		*Chlorella pyrenoidosa*	7	30.9%	1.41 g/L	Peng et al., 2020

**Main parameters**	**Factors**	**Microalgae type**	**Wastewater type**	**Lipid content**	**Biomass Growth/Productivity**	

Microalgae cultivation systems	Agricultural wastewater	*Chlorella sorokiniana*	BBM	–	0.25/d	Khalid et al., 2019
		*Chlorella sorokiniana*	POME	–	0.23/d	
		*Scenedesmus* sp.	Diluted swine w	17.19%	0.40 g/L	Zhao et al., 2022
	Industrial wastewater	*Chlorella vulgaris*	Tofu wastewater	23.25%	0.8 g/L	Dianursanti et al., 2014

**Lipid induction technologies**	**Factors**	**Microalgae type**	**Optimum conditions**	**Lipid intensity/Productivity**	**Effects of variation on lipid productivity/content**	

	Nutrient starvation stress	*Chloroidium* i	2.9 mM Nitrates	Lipid productivity 3.31 ± 0.16 mg/L/d	Lipid productivity 4.73 ± 0.12 mg/L/d, 40.37 ± 1.01% increase in lipid yield L/day	Prasanthkumar et al., 2020
		*Chloroidium saccharophillum*	0.5 mM Phosphate	Lipid productivity 2.31 ± 0.62 mg/L/d	Lipid productivity 3.96 ± 1.04 mg/L/d, 33.57 ± 3.72% increase in lipid yield L/day	
	Salinity stress	*Chloroidium saccharophillum*	10 mM NaCl	Lipid productivity 1.78 ± 0.32 Mg/L/d	Lipid productivity 4.70 ± 0.24 mg/L/d, 40.18 ± 1.97% increase in lipid yield L/day	Prasanthkumar et al., 2020
		*Chromochloris zofingiensis*	0.25 M NaCl	Lipid content 13 mg/g DW	Lipid content 195 mg/g DW, 15-fold increase in TAG content	Yaping et al., 2020
	Light stress	*Chromochloris zofingiensis*	400 μE/m⋅s	Lipid content 13 mg/g DW	Lipid content 195 mg/g DW, 15-fold increase in TAG content	Yaping et al., 2020
		*Chlorella* sp.	40 μmol/m⋅s	–	Higher lipid content of 17.2% achieved	Moreno-Garcia et al., 2019
	Temperature stress	*Gracilariopsis lemaneiformis*	High temperature 33 °C	Lipid intensity 5 × E10	Lipid intensity 7 × E10, Increase in C20:5 fatty acid by 88%, PUFA improved	Zhang X. et al., 2020
		*Porosira glacialis*	2 °C	Lipid content 19.5%	Lipid content peaked at 33.4 ± 4.0%	Jon et al., 2019
	Carbon dioxide supplementation	*Nannochloropsis* sp.	5% CO_2_ concentration	Lipid production 112.91 ± 17.34	Maximum lipid production of 782.7 ± 24.49 mg⋅L− 1 attained	Peng et al., 2020
		Arthrospira ZJU9000	171.2 mM HCO_3_^–1^ Concentration	Lipid content 9.5 (Wt%)	3.8-fold higher expression of gene encoding ATP-synthase.	Hongxiang et al., 2019

**Microalgae harvesting**	**Technique**	**Microalgae type**	**Operating conditions**		**Recovery efficiency**	

	Chemical flocculation (Self-flocculation)	*Chlorella vulgaris*	pH adjustment, NaOH; V = 30 L, pH = 10.8, HM = batch, CD = 9 mg/g		98%	Dries et al., 2011
			pH adjustment, Ca(OH)2,		98%	
			Ca(OH)2, V = 30 L; pH = 10.8; HM = batch, CD = 18 mg/g		
			pH adjustment, Mg(OH)2, V = 30 L; pH = 9.7; HM = batch, CD = 27 mg/g	98%	
		*Chlorella* sp.	P: Polyacrylamide; C: High; S: 1000; FD: 34; T: 60	98%	Arivalagan et al., 2019
		*Chlorella vulgaris*	P: Polyacrylamide; MW: High; C: High; D: 0.26; S: 3000; FD: 5; T: 30	100%	
	Bio-flocculation	*Chlorella* sp.	pH = 8, chitosan concentration: 100 mg/500 mL, cationic inducer concentration: 50 mg/L, stirring speed: 240 rpm	96.12%	Hadiyanto et al., 2022
		*Chlorella pyrenoidosa*	Citrobacter W4; bacterial-algal ratio: 4:1, G value: 26.30 s^–1^, harvesting time: 6 h	87.37 ± 2.96%	He et al., 2022
	Flotation	*Asterionella formosa*	Aluminum sulfate: 0.0314 ng Al/cell, pH = 7, Flotation time: 10 min	98.9%	Henderson et al., 2008a
		*Scenedesmus quadricauda*	CTAB: 40 mg/L, pH = 8, Flotation time: 20 min	90%	Chen et al., 1998
		*Chlorella vulgaris*	CTAB: 0.005 mequiv/L, pH = 7, Flotation time: 10 min	54%	Henderson et al., 2008b
		Chlorella	Ferric sulfate: 3.5 mg Fe/L, pH = 5.5, Flotation time: 10 min	94%	Jarvis et al., 2009
	Filtration	*M. aeruginosa*	CPAM-assisted filtration with a 100-μm sieve	90%	Cai et al., 2022
		*Chlorella vulgaris*	CF: 78 L/m^2^ h, F: 3 HZ	68.8%	Zhao et al., 2020
	Centrifugation	*Chlorella* sp.	Disk-stack centrifuge: 15 min	93%	Abhishek et al., 2016
		*Chlorella vulgaris*	Decanter centrifuges, Energy consumption: 8 kWh/m3, Final slurry Concentration: 22.2%	92%	Wiley and Campbell, 2011

#### Light

Generally, photosynthesis reaction comprises light reaction and dark reaction. In the light reaction phase, light energy is captured and converted into an energy carrier and then oxygen is released as a by-product (Sung et al., 2022). The electrons provided by oxygen can be transferred to photosystem I (PSI) and photosystem II (PSII) (Ranadheer et al., 2019). High-intensity light can lead to the photo-oxidation of PSII components, reducing the productivity of microalgae (Arun et al., 2021). While, low intensity light is not sufficient for the growth of microalgae. Therefore, an ideal light intensity is one of the essential parameters of the growth of microalgae, and microalgae usually absorb and use natural light with wavelengths ranging from 400 to 700 nm (∼43% of solar energy) (Teo et al., 2014).

#### Temperature

Temperature is an important parameter affecting microalgal growth. High temperature promotes the uptake and fixation of CO_2_ by microalgae, while excessively high temperature inhibits the respiratory metabolism of microalgae (Barten et al., 2021). The optimum growth temperature varies for different species of microalgae, but typically ranges from 20–30°C. Some freshwater microalgae can adapt to a broad range of temperature (5–35°C); however, in large-scale culture, the temperature must be restored to the ideal range (25–30°C) (Zhong et al., 2022). Therefore, temperature should be maintained at the optimal state for microalgal growth to prevent adverse effects on the metabolism of microalgae cells and reduction of lipid accumulation.

#### Salinity, nutrients, and pH

The optimum conditions for salinity, nutrients, and pH depend on the type of microalgae. Optimum salinity conditions are required for the healthy growth of microalgae. Salinity in the culture medium can be increased by adding NaCl and Na_2_SO_4_, while high salinity usually inhibits microalgal growth. Different species of microalgae have different salinity requirements. For instance, marine microalgae can tolerate higher salinity condition than freshwater microalgae (Maity and Mallick, 2022).

In addition, various nutrients are crucial for the growth of microalgae, including C, O_2_, H_2_, N, K, Mg, Ca, Fe, S, and P. The most important nutrients are C, O_2_, H_2_, N, P, and K, with C, O_2_, and H_2_ obtained from water and air and N, P, and K acquired from the culture medium (Chew et al., 2018; Wang et al., 2018). The main nutrient elements required for the growth of microalgae are N and P, and special types of microalgae also need some specific elements, such as Si element for *Diatoms*.

The pH in the culture medium also plays an important role in microalgal growth, which regulates enzyme activity, phosphorus availability, ammonia toxicity, and inorganic carbon availability. As microalgae grow, the pH gradually increases, and the culture medium becomes alkaline. As photosynthesis intensifies, OH^–^ ions gradually accumulate and microalgae readily capture CO_2_ from the atmosphere for conversion to biomass (Isiramen et al., 2022). Conversely, changes in pH affect the permeability of microalgae cell and the form of hydrated hydrogen ions of inorganic compounds.

#### Cultivation modes

In autotrophic mode, microalgae utilizes light and inorganic materials (CO_2_, water, and inorganic salts) for photosynthesis and synthesize organic materials (Ruiz et al., 2022). In heterotrophic mode, microalgae utilizes external carbon sources to grow in a dark environment. In microalgae mode, microalgae is provided with both CO_2_ and organic carbon sources. Compared with those in autotrophic mode, the yields of biomass and lipid in mixotrophic mode were significantly higher. In mixotrophic mode, the cell division of microalgae is also rapid. CO_2_ is one of the factors influencing the photosynthesis of microalgae; the increase in CO_2_ is beneficial for the improvement of the photosynthetic efficiency of microalgae, thereby increasing their biomass yield (Cheng et al., 2022).

### Lipid accumulation

The biological activity of microalgae is highly dependent on the culture conditions, and nutrients, carbon source, salinity, light, temperature, and metal ions all impact the lipid accumulation (Ma et al., 2022).

#### Nutrients

Nitrogen (N) is an important element impacting the cell structure of microalgae because it is essential in the synthesis of proteins, amino acids, nucleic acids, enzymes, and photosynthetic pigments (Gao et al., 2019). Phosphorus (P) is one of the most important nutritional elements that regulate cell growth and metabolism. It plays an important role in most cellular processes, especially in energy transfer, signal transduction, biosynthesis of macromolecules, photosynthesis, and respiration processes (Wu et al., 2021). Phosphatase converts organic phosphates into orthophosphate on the cell surface and microalgae take up phosphorus in the form of inorganic phosphate. In the presence of sufficient phosphorus, microalgae take up excessive phosphorus in the form of polyphosphate particles, and they can grow in the absence of phosphorus using stored phosphorus (Zhang et al., 2021).

#### Salinity

Salinity significantly affects the lipid accumulation in microalgae, which not only influences its growth but also resists infection by microorganisms such as bacteria. High salinity leads to increased intracellular osmotic pressure, entailing responses from physiological and biochemical mechanisms (Yang et al., 2022). High salinity impacts the fluidity and permeability of the microalgal cell membrane, under which the entry and exit of ions can be regulated through the cell membrane, and the accumulation of osmoprotective solutes as well as stress proteins becomes active. Salinity affects the cell structure and metabolic functions of microalgae and promotes the accumulation of lipid in microalgae (Zafar et al., 2021).

#### Metal ions

Trace metal elements have significant effects on the growth rate, as well as the accumulation of lipids and carbohydrates in microalgae. The addition of bioavailable Fe^3+^ to the culture medium can increase the growth rate and lipid accumulation in microalgae (Abd El Baky et al., 2012). While both excessively high and low levels inhibit them. High concentration of Mg^2+^ can enhance the activity of acetyl coenzyme A carboxylase (ACCase), thus promoting lipid accumulation (Qian et al., 2021). Ca^2+^ is more critical in the signal transduction to regulate the synthesis of lipids in microalgae (Di Caprio et al., 2018). Heavy metals such as Cd, Cu, and Zn can alter the lipid metabolism in microalgae and promote lipid accumulation (Urrutia et al., 2019).

#### Temperature

Seasonal temperature fluctuations, daily temperature changes, and sudden temperature changes due to various reasons can unavoidably change the growth conditions of microalgae, thus affecting its growth efficiency (Xin et al., 2011). Temperature changes impact the metabolic pathways of microalgae, with both high and low temperature stresses promoting lipid accumulation in microalgae (Rehman et al., 2022).

#### Light

Microalgae is photosynthetic organism and light is essential for its growth. However, microalgae can only absorb a small fraction of the natural light with a wavelength range of 400–700 nm (∼43% of solar energy) (Yan et al., 2016). So light with the appropriate wavelength and intensity is the key factor that influences or even controls the growth and lipid accumulation of microalgae.

### Microalgae harvesting

Microalgae harvesting, the separation of microalgae from the culture medium, is the main problem in the downstream process of microalgal biodiesel production. Excessively high microalgal concentration leads to overshadowing and results in insufficient light and consequently lower productivity. Hence, the biomass concentration of microalgae in culture medium is generally maintained at a low level (0.5–5 g/L), which means that a large amount of water needs be removed to harvest microalgae (Liu et al., 2018). Microalgae harvesting is further complicated by their small cell size (2–20 μm) and stable colloid in the suspension. Hence, the sedimentation efficiency of microalgae cell is a function of the microalgae cell size, cation concentration in the culture medium, and extracellular secretions of the microalgal cell (Ortiz et al., 2021).

#### Cell size

Currently, the studies on flocculation of microalgae focus on single species cultured under specific conditions. Flocculation depends mainly on the surface properties of the microalgae cell, which vary according to the species and culture conditions. The specific surface area of the microalgal cell increases as the cell size decreases (Min et al., 2022). Therefore, the smaller the microalgae cell, the higher the flocculant dose required. Meanwhile, relatively large microalgae cell favors the sedimentation process.

#### Cation concentration

Divalent cations (such as Ca^2+^ and Mg^2+^) and trivalent cations (such as Al^3+^ and Fe^3+^) influence the flocculation efficiency of microalgae. The latter attach to the negatively charged carboxyl groups on the surface of microalgae and form flocculates through charge neutralization, while the former mainly bind to the negatively charged extracellular polymeric substance (EPS) on the microalgal surface cells to promote their self-flocculation (Choi et al., 2020).

#### pH

pH is an important parameter that needs to be carefully considered. The pH affects the charge on the surface of the microalgae cell and also impacts the charge of chemical flocculants. The negative charge on the surface of the microalgae cell gradually decreases as the pH increases. When the pH exceeds 10, the surface of microalgae cell approaches “zero charge,” and the cell transfers from a homogeneous stable phase to an inhomogeneous unstable phase, leading to their flocculation and sedimentation (Liu J. et al., 2014). However, Mg(OH)_2_, CaCO_3_, and Ca_3_(PO_4_)_2_ also precipitate under higher pH.

#### CO_2_

Microalgae consumes CO_2_ through photosynthesis resulting in elevated pH, which could cause self-flocculation. Most commercial microalgae culture units typically rely on CO_2_ to enhance the yield of microalgae. The use of CO_2_ in controlling microalgal self-flocculation is a promising microalgal technology, and the CO_2_ concentration mechanism (CCM) of microalgae is an important determinant of the effectiveness of microalgae absorption of HCO_3_- (Liu G. et al., 2014). Most microalgae have a highly active CO_2_ concentration mechanism and can sustain photosynthesis as the pH exceeds 10, which in turn causes microalgae self-flocculation (Tran et al., 2017).

#### Extracellular polymeric substance

Flocculation of microalgae is usually accompanied by the production of EPS, which mainly contain proteins, polysaccharides, humic substances, and nucleic acids. In fact, EPS play a crucial role in the flocculation of microalgal cell, which acts as a bridge to connect microalgal cell and produce large aggregates, a phenomenon term as self-flocculation. In addition, extracellular secretions of microalgal cell and metal cations in the culture medium can interact to promote sedimentation of microalgae (Wang et al., 2022). The positively charged metal cations combine with the negatively charged carboxyl groups in the cell wall and the carboxyl and hydroxyl groups in the extracellular secretion to form flocs. The production of extracellular secretions by microalgae is a function of nutrient content, growth stage of microalgae, and external environment (Taghavijeloudar et al., 2021).

### Lipid extraction

Microalgae cell wall mainly comprise proteins, cellulose, hemicellulose, and pectin. They are thick and hard, and represent the main challenge for microalgae lipid extraction. The mechanical strength and thickness of microalgae cell wall dictate the ability of microalgae cell to resist mechanical fragmentation (e.g., homogenization under high pressure). Furthermore, the properties of the microalgae cell wall are impacted by external conditions such as salinity, nutrients, and light.

#### Salinity

Munns et al. (1983) found that when microalgae grown in a high salinity culture medium, the cell wall of *Chlorella* accounted for up to 70% of the total cell, suggesting that cell wall thickness varied according to the microalgae culture conditions. The building block of their cell wall is cellulose, and the enzymes associated with cellulose synthesis are strongly influenced by salinity (Menegazzo and Fonseca, 2019). However, the effect of salinity on the cell wall components and mechanical strength of microalgae has not been studied.

#### Nutrients

Although the strength and elasticity of the cell walls of various microalgal species differ, the cell size continues to be an important determinant of cell resistance to rupture in high-pressure homogenizers. It might be the stress response from N-deficient microalgae cells to enhance the resistance to rupture. In addition to thicker cell walls, their average size was also larger than that of N sufficient microalgae cells (Enamala et al., 2018). In N-deficient microalgae cells, cell enlargement increased the susceptibility to cell fragmentation. In addition to cell size and cell wall thickness, factors such as the composition of the cell wall may also contribute to the enhanced susceptibility of microalgae to mechanical fragmentation (Ahmed and Kumar, 2022).

#### Light

Short-wavelength ultraviolet B (UV-B) and ultraviolet C (UV-C) rays are the most destructive and cause cellular damage. Ultraviolet A (UV-A) is less effective and indirectly damages cells by generating reactive oxygen species that may damage DNA, proteins, and lipids (Kuluncsics et al., 1999). UV-B and UV-C cause direct DNA damage through the absorption of photons by DNA bases, causing chemical quenching and the formation of pyridine dimers in the sequence (Rastogi et al., 2010). UV-induced cell lysis is attributed to the uptake of UV radiation by DNA, RNA, proteins, and lipids, resulting in structural damage and signaling/metabolic disruption in the cell. Upon exposure to natural light, DNA damage in algal cells can undergo photo repair through the enzyme cyclobutane pyrimidine dimer photolyase (Rastogi et al., 2010). Microalgae are usually stored in the dark after UV irradiation to reduce photo repair and thus maximize cell damage, as this repair mechanism prevents cell lysis. On an industrial scale, the UV radiation of sufficient intensity can irreparably damage microalgae. UV-B radiation causes the formation of pyrimidine dimers in DNA, which can lead to mutations in the cellular genome. In algal cells, if severe mutation occurs, the cells may lyse upon necrosis or apoptosis, leading to the uncontrollable release of intracellular components. This is also commonly referred to as programmed cell death because of its highly regulated nature and is activated if sufficient DNA mutations occur in specific genes. During apoptosis, cell “package” intracellular components, including lipids, into apoptotic bodies that are released into the culture medium (Sydney et al., 2018).

## Technologies for microalgae biodiesel production

With advances in microalgae biodiesel production research, the development of microalgae cultivation systems, lipid induction, microalgae harvesting, and lipid extraction technology have become the hot topics (Chhandama et al., 2021).

### Microalgae cultivation systems

Microalgae can grow in different aquatic environments, such as freshwater, seawater, industrial wastewater, agricultural wastewater etc., as long as there are adequate amounts of carbon element (organic or inorganic), nitrogen element (urea, ammonia, or nitrate), phosphorus, and other trace elements (Purba et al., 2022). The utilization of wastewater in large-scale microalgae cultivation for biodiesel production has a vast potential. However, considerable uncertainty and challenges remain in microalgae cultivation using wastewater (Cheah et al., 2016), including changes in wastewater compositions caused by the source, infrastructure, weather conditions, and preprocessing methods; uneven nutrient ratios (e.g., those of N/P and C/N); light transmission affected by high turbidity; presence of competing microorganisms and toxic compounds; and deterioration caused by water recycling and reuse, which leads to the accumulation of growth-inhibiting substances. At present, the utilization of domestic wastewater, agricultural wastewater, and industrial wastewater for microalgae cultivation has been widely studied. The comparison of the microalgae cultivation systems is in Table 2.

#### Agricultural wastewater

A comparison of the mineral composition between livestock wastewater and microalgae culture medium found that the former is an ideal culture medium for microalgae growth (Wang and Zhang, 2022). In the growth, microalgae can effectively remove N and P from the livestock wastewater. As the nutrient uptake efficiency of some benthic microalgae is significantly higher than that of planktonic microalgae, benthic microalgae are more effective in uptake nutrients from pig manure wastewater (Zhou et al., 2022). However, many problems are still associated with the utilization of livestock wastewater for microalgae cultivation (Arutselvan et al., 2022): (a) The presence of a large amount of solid particles affects the light penetration in the wastewater; (b) Very high levels of nutrients, particularly NH_3_–N, inhibit microalgae growth; (c) A large amount of carbon source is locked in insoluble organic compounds, which make it difficult for microalgae to absorb; (d) A large volume of freshwater would be consumed to dilute livestock wastewater; and (e) Efficient microalgae strains that are adapted to the adverse environment of livestock wastewater have not yet been developed.

To solve these aforementioned problems, many methods and techniques have been developed and employed. Martin et al. (1985) suggested inoculating high concentrations of microalgae strains in diluted pig manure wastewater (diluted 20–100 times) as a way for treating pig manure wastewater and producing protein-rich microalgal biomass. After a suitable dilution, the NH_3_-N levels in the livestock wastewater is within the ideal range, which would not inhibit microalgae growth. However, the use of a high dilution factor lowers soluble organic and inorganic carbon levels, which are insufficient to maintain microalgae growth. The addition of exogenous carbon dioxide as an inorganic carbon source helps increase microalgae biomass yield and pollutants removal efficiency. When wastewater with high organic matter content (such as pig and cow manure wastewater) is used for microalgae culture, the trophic mode of the microalgae (autotrophic, heterotrophic, and mixotrophic) is critical in determining the microalgae biomass yield and pollutants removal efficiency.

In summary, screening mixotrophic microalgae strains that are well adapted to wastewater environments, developing preprocessing methods that can effectively improve volatile fatty acid composition, and developing efficient culture systems are methods with the greatest potential for treating livestock wastewater and maximizing microalgae biomass yield.

#### Industrial wastewater

Currently, research topics in the utilization of industrial wastewater for microalgae culture are focused on the removal of heavy metal pollutants (Cd, Cr, Zn, etc.) and organic toxins (hydrocarbons, biological bactericides, and surfactants) from industrial wastewater and not on biomass accumulation and biodiesel production (Japar et al., 2021). Recently, a microalgae cultivation technique was developed to treat wastewater in oil production. The data from United States Department of Energy shows that 56 million barrels of wastewater are produced every day during the onshore production of petroleum and natural gas in the United States. If this wastewater is used for microalgae culture, and microalgae can reach 1 g/L biomass concentration and 30% lipid content, 700,000 gallons of biodiesel can be produced every day, which accounts for a large part of the existing transportation fuel needs. Notably, the wastewater from the food-processing industry, such as the wastewater from the processing of olive oil and molasses, is in a different category from industrial wastewater, as it lacks heavy metal pollutants and toxic organic matters and is very suitable for microalgae cultivation (Sirohi et al., 2022).

In summary, the utilization of wastewater for microalgae cultivation not only reduces the addition of nutrient, but also decreases the concentrations of nitrogen, phosphorus, and other pollutants in wastewater, which is the main method for decreasing the cost of microalgae cultivation. However, there is no “ideal wastewater” that can provide nutrients similar to artificial culture medium and is suitable for microalgae growth to achieve maximum biomass yield and effective pollutants removal. In this technology, wastewater dilution, sterilization, and oxidation would consume large amounts of freshwater resources and energy.

### Lipid induction technologies

To improve the lipid yield of microalgae, effective lipid induction techniques, such as nutrient starvation, salinity stress, light stress, temperature stress, and metal stress, were developed. The comparison of these technologies is in Table 2.

#### Nutrient starvation stress

Nutrient starvation is an economically feasible and environmentally friendly method to increase the yield of lipid in microalgae efficiently. So far, nutrient starvation has been the most successful and widely used technique for lipid induction, and N, P, and S starvation are the most widely used methods for lipid induction of microalgae. Large amounts of lipids in microalgae are produced under N stress, while the amino acid content is significantly decreased (Breuer et al., 2012). Lipid content increased from 10 to 29.5% when *Scenedesmus* was cultured under P stress. S stress can regulate the distribution of carbon element in microalgae to promote the lipid accumulation. Except for N, P, and S starvation, the consumption of nutrient elements during microalgae cultivation is another effective starvation technique for lipid production, because all elements can achieve starvation in the culture medium (Srinuanpan et al., 2018). Carbon fixed through photosynthesis can be used for the synthesis of lipids, proteins, and carbohydrates. N is essential for the protein synthesis, and the limiting N concentration affects cell division and photosynthesis and decreases the growth rate of microalgae cell (Wang et al., 2015). N starvation also affects carbon distribution in microalgae. When N element is sufficient, carbon fixed through photosynthesis is 7–10 times that of the N assimilation rate, which is adequate for the synthesis of N-containing cellular components, such as proteins, nucleotides, and pigments (Su, 2021).

#### Salinity stress

Salinity can affect various biochemical reactions associated with growth and metabolism in microalgae. High salinity will impede the absorption of water and nutrient by microalgae, thereby inhibiting growth and ultimately leading to the death of microalgae. There are three types of salinity stress: ionic stress, osmotic stress, and oxidative stress. Ionic stress is caused by ionic homeostatic imbalance. Under salinity stress, the competition between Na^+^ and K^+^ causes K^+^ deficiency in the cytoplasm. Simultaneously, salinity stress also causes an imbalance in reactive oxygen species as well as oxidative stress. In microalgae cells, oxidative stress is often accompanied by an increase in lipid yield. Culturing microalgae under salinity stress can prevent contamination by pollutants and competition by invasive species and other microorganisms (Chen et al., 2022). However, very high salinity can inhibit the growth and change the morphology of microalgae cell (Zafar et al., 2021). Therefore, an ideal salinity range must be determined.

#### Light stress

Microalgae growth requires light. A strong light intensity is beneficial for lipid accumulation in microalgae cell, because it favors storage of excessive photosynthetic products and further converts them to chemical energy. However, the light intensity required to achieve the highest lipid yield differs based on microalgae species, as their light utilization efficiency is different by different microalgae species (Gao et al., 2022). Limiting or saturated light intensity are not favorable for microalgae growth. At a very low light intensity (below a compensation point), microalgae growth is affected, which also has adverse effects on lipid accumulation (Aburai et al., 2021). Conversely, after the compensation point is reached, microalgae yield increases with increasing light intensity, and photosynthetic efficiency is the highest at the saturation point. Therefore, an increase in light intensity has a positive effect on increasing lipid accumulation in microalgae. However, very high light intensity will cause photoinhibition, thereby decreasing lipid accumulation in microalgae (Sousa et al., 2012).

#### Temperature stress

The effects of temperature are similar to light intensity on microalgae growth and lipid accumulation. Both increase exponentially as temperature increases and can reach a maximum. However, the required temperature for maximum yield of microalgae also differs by microalgae species. The lipid content in *Chlorella* reaches a maximum at 25°C, and significantly decreases as temperature decreases (Converti et al., 2009). When the temperature increases from 20 to 27.5°C, lipid content in *Scenedesmus obliquus* increases from 18 to 40% (Vitova et al., 2015). However, not all lipid content increases with increasing temperature.

#### Metal stress

Metal ions play a vital role in microalgae growth and lipid accumulation. Metals such as iron and copper are components of photosynthetic electron transport proteins. However, some metals like cadmium, chromium, lead, mercury and arsenic could cause negative effects (like impairing photosynthetic mechanism, blocking cell division and inhibiting enzyme activity) in algal cells. *Chlorella* has a strong resistance to copper and cadmium ions under heterotrophic conditions, with lipid yield increasing by 93.9 and 21.1%, respectively (Yang et al., 2015). In addition, Mg^2+^ is also found to affect the cell growth and lipid production in microalgae (Ren et al., 2014). Mg^2+^ plays an important part in different metabolic processes and physiological pathways of algae and is a major element in microalgae culture medium. Mg^2+^ is a component of chlorophyll molecules, and Mg^2+^ level in the culture medium directly affects the bio-formation of chlorophyll. It has been reported that the increase of Mg^2+^ level could enhance the acetyl-CoA carboxylase (ACCase) *in vivo* activity, and ACCase exerted intense regulation of fatty acid synthesis in microalgae.

#### Carbon dioxide supplementation

Carbon dioxide ensures a carbon supply for photosynthesis in photosynthetic microalgae, as microalgae growth requires sufficient soluble carbon dioxide. Microalgae content and lipid yield increases when carbon dioxide increases to an optimal level. Limited carbon dioxide content in the culture medium can decrease the metabolic rate, thereby decreasing lipid yield in microalgae (Aghaalipour et al., 2020). Therefore, a high concentration of carbon dioxide can satisfy the carbon needs of microalgae. To decrease the costs, flue gas (rich in carbon dioxide) can be introduced as a carbon source into the microalgae culture system (Mousavi et al., 2022). However, very high carbon dioxide concentration can negatively affect microalgae growth because unused carbon dioxide is converted to carbonic acid (H_2_CO_3_) and decrease the pH of the culture medium. Therefore, an optimal carbon dioxide concentration is needed to maximize the lipid yield in microalgae.

### Microalgae harvesting technologies

Techniques for microalgae harvesting include centrifugation, coagulation, flocculation, filtration, and centrifugational or gravitational sedimentation. Microalgae harvesting is usually divided into two stages: mass harvesting and thickening. The objective of mass harvesting is to separate microalgae from the suspension and obtain 2–7% of solid mass through flocculation, foam flotation, or gravitational sedimentation (Fuad et al., 2018). The objective for thickening is to enrich biomass through filtration and centrifugation, and this process requires higher energy input than mass harvesting (Ortiz et al., 2022). The choice of harvesting methods is mainly determined by the characteristics of microalgae, such as cell density and size. The comparison of these technologies is in Table 2.

#### Chemical flocculation

During the chemical flocculation, chemicals are used to aggregate microalgae cells to induce flocculation. Microalgae cell is negatively charged and repel each other, resulting in the cell suspension, and the introduction of chemical substances can neutralize the charge and cause cell aggregation (Pittman et al., 2011). Coagulants are mainly classified as inorganic and organic coagulants. Inorganic coagulants, such as iron and aluminum salt coagulants, are used to harvest microalgae, such as *Scenedesmus* and *Chlorella.* Organic coagulants, such as chitosan, are biopolymers that can increase microalgae dimensions (>100 μm), thereby increasing their sedimentation efficiency. Currently, cation coagulants are optimal for harvesting microalgae, and anions cannot be used for microalgae flocculation due to repulsion between the charges.

#### Biological flocculation

Biological flocculation is a method that uses extracellular polymers secreted by bacteria or fungi or surface charges on these microorganisms to flocculate microalgae. Compared with other flocculation methods, biological flocculation is a cheap and environmentally friendly method that can be continuously used for large-scale microalgae harvesting. Biological flocculation is affected by pH, temperature, nutrient content, and dissolved oxygen. However, when bacteria are used to aid microalgae flocculation harvesting, additional nutrients need to be added, which may result in a secondary contamination (Wan et al., 2015).

#### Self-flocculation

Self-flocculation refers to the use of cell surface characteristics or other factors to cause microalgae aggregation. Natural self-flocculation of microalgae may be caused due to environmental stress and changes in N levels, pH, dissolved oxygen content, and Ca^2+^ and Mg^2+^ levels in the culture medium (Enamala et al., 2018). Self-flocculation usually occurs naturally after carbon dioxide is exhausted or in basic pH conditions. Mainly Ca and Mg precipitates are formed as a consequence of natural self-flocculation.

#### Flotation

Flotation refers to the use of microbubbles to harvest microalgae without the addition of chemicals. During the flotation, air bubbles (diameter < 500 μm) bind to the microalgae cells. Based on air bubble dimension, flotation can be divided into dissolved air and dispersed flotation. In dissolved air flotation, small air bubbles with a mean size of 40 μm (range: 10–100 μm) are generated (Laamanen et al., 2016). Flotation processes are classified according to the method of bubble production as dissolved air flotation (DAF), dispersed air flotation (DiAF), electrolytic flotation, and dissolved ozone flotation (DOF). Sometimes, dissolved air flotation uses flocculants and compressed bubbles to accelerate the aggregation of microalgae, which float to the water surface (Alkarawi et al., 2018). This is an efficient method for harvesting microalgae, but high-pressure requirements result in higher operating costs.

#### Sedimentation

Sedimentation is commonly used for separating microalgae from water. The density, shape and diameter of microalgae cells are the main factors affecting the velocity of microalgae sedimentation. Lamella settler and settling ponds can increase the harvest efficiency of microalgae. However, cell density is a limiting factors as high density microalgae cells are easier to harvest through gravitational sedimentation than low density microalgae cells. Sedimentation is a conventional separation technique that has been widely used in water treatment to remove unwanted particles in a cost-effective manner. In this process, particles are left to settle down according to Stokes’ Law (Shelef et al., 1984). In theory, the settling velocity has been reported to be 0.4 and 2.2 m/day. This is because Stokes’ law is considered for the spherical-shaped cells, whereas most microalgae strains in nature have complex morphology (Peperzak et al., 2003).

#### Filtration

Traditional filtration is used to harvest larger microalgae cells (usually > 70 mm). While microfiltration, ultrafiltration and reverse osmosis can be used to harvest smaller microalgae, and these filtration methods require periodic replacement of expensive membranes, resulting in high costs. Based on the configuration of a feeding flux during a filtration process, there are three types of filtrations, including dead-end filtration (DEF), cross-flow filtration (CFF), and dynamic filtration (DF). Dead-end filtration, also known as frontal filtration or cake filtration, is the most common membrane system in microalgae harvesting. They are particularly effective in recovering microalgal cells with a radius larger than 70 μm (Barros et al., 2015). Cross-flow filtration (tangential flow filtration)–a filtration process in which feed flows tangentially across a membrane surface–is widely utilized in microalgae harvesting depending on the process conditions (Ahmad et al., 2012). Cross filtration works by introducing feed flow under pressure across the membrane surface instead of directly onto the filter. During filtration, any material smaller than the cross-flow membrane pore passes through the membrane, while larger suspended particulates remain in the retentate stream, similar to DEF. Dynamic filtration, also known as shear enhanced filtration, is considered an alternative option to DEF and CFF. Due to fouling in DEF, transmembrane pressure (TMP-the pressure needed to press the flux through a membrane) increases with the formation of the cake layer, resulting in lower filtration efficiency (Cheng et al., 2021). To address this issue, the mechanical movement of devices is applied in DF that creates high shear stress at the membrane surface, resulting in uncoupling between local shear rate and TMP from a feeding flowrate, which leads to higher efficiency compared to the conventional filtration methods.

#### Centrifugation

Centrifugation uses centrifugal force to separate mixtures and is widely used to harvest microalgae, which produces at maximum yield of 95% under optimal conditions. Centrifugation is energy-intensive (with a range varying from 0.3 to 8 kWh/m^3^) due to its high capital and operating costs (Najjar and Abu-Shamleh, 2020). Centrifugation takes up to 20-30% of the total costs in biofuel production, and these costs are largely associated with the dewatering process (drying of microalgae suspension). In other words, even though the size of cells has a direct relationship with the required g-force, but the technique can still be used in the process. Among sedimenting centrifuges, three types are frequently used for algae separation on industrial scales: disk stack, nozzle-type, and decanters centrifuges (Sharma et al., 2014). Stationery-wall centrifuges (hydrocyclones) separate particles in a liquid suspension with no moving parts. There is also another family of centrifuges, namely benchtop centrifuges, which are commonly used in laboratories (Show and Lee, 2014). The limitations of this method are high operation and maintenance costs. Moreover, it is not suitable for large-scale application.

### Lipid extraction technologies

Lipids are extracted after the microalgae are harvested and lyophilized. The principles of lipid extraction from microalgae cell are specific—no disruption to lipid components and ease of scaling up. The lipid extraction process includes cell drying, cell disruption, and lipid extraction. Solvent extraction and supercritical fluid extraction are the common techniques used for extracting lipids from microalgae cell. The Folch method was previously used for lipid extraction from microalgae cell due to its simple operation (Karim et al., 2020). However, this method requires a large volume of solvent and tends to be affected by mineral salts—in the absence of which acidic lipids in microalgae are washed away—in the material used for extraction. Therefore, the modified Bligh & Dyer method is widely used for lipid extraction. The solvents used for lipid extraction should be cheap, non-volatile, non-toxic, and non-polar (Abimbola et al., 2021). Before lipid extraction, the cell wall and membrane of microalgae must be disrupted. This is because the microalgae cell walls are thick and tough and can protect cells from degradation due to the lipid extraction conditions, affecting efficiency.

#### Preprocessing: Cell disruption

Microalgae must undergo disruption before lipid extraction. The cell disruption methods are determined by the type, status, and scale of the microalgae and divided into mechanical and non-mechanical preprocessing methods (Li et al., 2022).

##### Mechanical preprocessing

###### Milling

Milling uses shear force to collide microalgae cell with glass or ceramic beads in the stirrer to disrupt microalgae cell. When the bead size is <0.5 mm or >0.5 mm, increasing bead size can either promote or have adverse effects on algal cell disruption, respectively (Zinkoné et al., 2018). Additionally, high density (zirconium) and low density (glass) beads are more effective in high- and low-viscosity media, respectively. Increasing stirring duration, stirring speed, and bead quantity has a positive effect on disruption (Liu et al., 2022). Although milling has many advantages, one of its limitations is its high energy consumption. Moreover, the formation of very fine cell debris and biochemical substances in the soluble and solid phases increases downstream processing cost. Therefore, milling is not an ideal cell disruption method.

###### High-pressure homogenization

High-pressure homogenizati on uses the high-pressure impact (shear force) of an accelerated fluid jet on the surface of a stationary valve and hydraulic cavitation due to shear stress induced by pressure decrease to achieve cell disruption. Cavitation is a three-step phenomenon that occurs within a short time interval (microseconds): air bubble formation, growth, and rupture. This leads to the release of large amounts of energy that causes cell disruption (Carullo et al., 2018). Cell density must be low when using high-pressure homogenization to disrupt microalgae cell, and the products must be separated. It increases energy and water consumptions for downstream processing (Spiden et al., 2013).

###### Ultrasonication

In ultrasonication processing, the energy of high-frequency sound waves causes cavitation, and the transmitted impact waves form jets in the surrounding medium, causing cell disruption through high shear-force. The advantages of this technique are that the cell walls are disrupted at low temperatures and no additional of chemicals are involved. The main disadvantages of ultrasonication are low disruption rate of some types of microalgae and local or global heat generation (Gerde et al., 2012). However, good temperature control can increase the quality of extracted lipid while decreasing the disruption rate of microalgae cell. Combining ultrasonication and solvent use or other disruption methods can increase cell disruption rate and decrease energy consumption.

##### Non-mechanical preprocessing

###### Biological enzyme

Biological enzyme has various unique characteristics, such as biological specificity, gentle operating conditions, low energy consumption, and destruction rates, etc. Biological enzyme has great potential in cell disruption. Glycoside hydrolase, glucanase, peptidase, and lipase have been used in the lysis of various microalgae. During lysis, the enzyme binds to specific molecules on the cell membrane or wall to destroy it. However, the treatment duration is long and disruption efficiency is lower than that of mechanical or chemical disruption methods (Li et al., 2022). Additionally, the main limiting factor for biological enzyme is the high cost and the low number types of enzymes available for microalgae cell disruption.

###### Chemical disruption

Many chemicals can disrupt microalgae cell, such as antibiotics, chelators, detergents, chemical solvents, hypochlorite, acids, and bases (Li et al., 2022). The selectivity, suitability, and efficiency of these chemicals are determined by the wall composition of microalgae cell. Chemicals differ in their mechanism of microalgae cell: For example, antibiotics usually inhibit the synthesis of microalgae cell membrane components, chelators can bind to cations that form molecular cross-linked bridges between neighboring microalgae cell membrane, detergents and membrane molecules form micelles, chemical solvents dissolve or perforate microalgae cell membranes or microalgae wall, bases can saponify membranes, and acids can cause microalgae cell membrane or microalgae cell wall perforation (Phong et al., 2018). However, the problems of efficiency, toxicity, and economical feasibility exist with chemical disruption methods.

#### Lipid extraction

The lipid in microalgae cell contains different components. Non-polar organic solvents can disrupt hydrophobic interactions between neutral lipids, whereas polar organic solvents, such as alcohols, can disrupt the hydrogen bonds between polar lipids. Solvent selection is based on the type of microalga and should be cheap and non-toxic. Soxhlet extraction, which uses hexane, and the Bligh & Dyer method, which uses a chloroform/methanol mixture, are the two most commonly used methods for extracting lipids from microalgae cell (Sanyal et al., 2022). Hexane is becoming increasingly popular for lipid extraction due to its low cost. Additionally, ionic liquids (ILs) have been successfully used for lipid extraction.

##### Soxhlet extraction

Soxhlet extraction method was proposed as early as 1879 and was originally used to quantify the total lipids amount in milk (Soxhlet, 1879) and was further gradually promoted in the fields of food, pharmaceutical and other industries. Hexane extraction can be used alone or in combination with homogenizers. After a homogenizer is used to extract lipids, a mixture of hexane and cyclohexane can be used to extract the lipids. Lipids dissolved in cyclohexane and microalgae residues are filtered out and then separated using distillation. The disadvantage of solvent extraction is that the chemicals used are more hazardous. For example, benzene is carcinogenic and explosive. Although hexane has a lower efficiency than chloroform, it has a lower toxicity and affinity toward non-lipid components, and a higher selectivity toward neutral lipid fractions (Aravind et al., 2021). Although the traditional Soxhlet extraction has low cost and simple operation, it also has disadvantages such as long extraction time and large reagent consumption.

##### Bligh and dyer extraction

The Bligh-Dyer extraction method is a method based on a two-phase solvent extraction and it can be considered as a variant of Folch (Bligh and Dyer, 1959). In the Bligh & Dyer extraction, the critical ratio of methanol, chloroform, and water should be 2:1:1.8, under which samples can be homogenized into a monophasic system. Chloroform is added for rehomogenization, and the final ratio of methanol, chloroform, and water is 2:2:1.8. The homogenate is centrifuged and two layers are formed (lipids dissolved in chloroform and methanol dissolved in water) and distillation is used to separate the lipids from chloroform and methanol (Candice et al., 2019). As a method close to Folch, Bligh-Dyer also uses toxic reagents, which generates a large amount of environmentally harmful waste. Therefore, waste recovery and cost need to be considered when it is used for large scale extraction.

##### Ionic liquid extraction

Ionic liquids are the salts composed of relatively large asymmetric organic cations and smaller inorganic or organic anions. Cations usually consist of nitrogen-containing ring structures (e.g., imidazopyrimidine or pyrimidine), including various functional groups, which determine the polarity of ILs. ILs are known as green solvents, because their non-volatile and heat-stable characteristics make them potential substitutes for volatile organic solvents (Shankar et al., 2017).

##### Supercritical fluid extraction

Supercritical fluid (such as water, CO_2_, etc.) extraction is a green technology with the potential to replace traditional organic solvent extraction (Jafari et al., 2021). Among the fluids, CO_2_ is very fashionable. The extraction apparatus includes a feeding pump used for compressing and transporting liquid carbon dioxide and a heated micro-metering valve. Once heated, the compressed carbon dioxide will be at a supercritical state and can extract lipids from microalgae. Once full decompression occurs, carbon dioxide evaporates into the surrounding environment and the extracted lipids are precipitated out. Supercritical carbon dioxide has high solvation capacity and low toxicity (Obeid et al., 2018). The intermediate diffusion or viscosity of the fluid promotes mass transfer equilibrium, and the extracted lipids do not contain solvents. The disadvantage of this method is the complex equipment required and high cost.

## Conclusion

Microalgae could utilize the carbon resource in the water and soil in the nature, and the biodiesel could be produced from microalgae, which provides a more sustainable and environmentally friendly alternative to fossil fuel. This review discusses the influencing parameters and advanced parameters technologies for microalgae biodiesel production. All key parameters affecting microalgae cultivation, lipid accumulation, microalgae harvesting, and lipid extraction were critically listed, and the recent advanced technologies for microalgae biodiesel production including microalgae cultivation systems, lipid induction technologies, microalgae harvesting technologies, and lipid extraction technologies were also compared. While, the use of microalgae as biodiesel feedstock is technically feasible, but not economically viable. The production of microalgae biodiesel in the form of a hybrid refinery along with production of conventional microalgae products can improve the marketability of microalgae. However, more research should be done on long term stability and technical aspects of a hybrid refinery.

## Author contributions

SZ, LZ, GX, FL, and XL: conceptualization, writing – review and editing, and resources. SZ and LZ: methodology. SZ: writing – original draft preparation. SZ, LZ, and XL: funding acquisition. LZ and XL: supervision. All authors contributed to the article and approved the submitted version.

## References

[B1] Abd El Baky H. H. El-Baroty G. S. Bouaid A. Martinez M. Aracil J. (2012). Enhancement of lipid accumulation in Scenedesmus obliquus by Optimizing CO2 and Fe3+ levels for biodiesel production. *Bioresour. Technol.* 119 429–432. 10.1016/j.biortech.2012.05.104 22727605

[B2] Abhishek G. Rohit M. Poonam S. Ismail R. Faizal B. (2016). An innovative electrochemical process to alleviate the challenges for harvesting of small size microalgae by using non-sacrificial carbon electrodes. *Algal Res.* 19 292–298. 10.1016/j.algal.2015.08.014

[B3] Abimbola T. Christodoulatos C. Lawal A. (2021). Performance and optimization studies of oil extraction from Nannochloropsis spp. and Scenedesmus obliquus. *J. Clean. Prod.* 311:127295. 10.1016/j.jclepro.2021.127295

[B4] Aburai N. Nishida A. Abe K. (2021). Aerial microalgae Coccomyxa simplex isolated from a low-temperature, low-light environment, and its biofilm growth and lipid accumulation. *Algal Res.* 60:102522. 10.1016/j.algal.2021.102522

[B5] Aghaalipour E. Akbulut A. Güllü G. (2020). Carbon dioxide capture with microalgae species in continuous gas-supplied closed cultivation systems. *Biochem. Eng. J.* 163:107741. 10.1016/j.bej.2020.107741

[B6] Ahmad A. L. Mat Yasin N. H. Derek C. J. C. Lim J. K. (2012). Crossflow microfiltration of microalgae biomass for biofuel production. *Desalination* 302 65–70. 10.1016/j.desal.2012.06.026

[B7] Ahmed J. Kumar V. (2022). Effect of high-pressure treatment on oscillatory rheology, particle size distribution and microstructure of microalgae Chlorella vulgaris and Arthrospira platensis. *Algal Res.* 62:102617. 10.1016/j.algal.2021.102617

[B8] Alkarawi M. A. S. Caldwell G. S. Lee J. G. M. (2018). Continuous harvesting of microalgae biomass using foam flotation. *Algal Res.* 36 125–138. 10.1016/j.algal.2018.10.018

[B9] Aravind S. Barik D. Ragupathi P. Vignesh G. (2021). Investigation on algae oil extraction from algae Spirogyra by Soxhlet extraction method. *Mater. Today Proc.* 43 308–313.

[B10] Arivalagan P. Sutha S. Peter B. Nándor N. Ao X. Rajesh B. J. (2019). A review on chemical mechanism of microalgae flocculation via polymers. *Biotechnol. Rep.* 21:e00302. 10.1016/j.btre.2018.e00302 30671358 PMC6328355

[B11] Arun S. Ramasamy S. Pakshirajan K. (2021). Mechanistic insights into nitrification by microalgae-bacterial consortia in a photo-sequencing batch reactor under different light intensities. *J. Clean. Prod.* 321:128752. 10.1016/j.jclepro.2021.128752

[B12] Arutselvan C. Seenivasan H. K. Lewis Oscar F. Ramya G. Thuy Lan, Chi N. (2022). Review on wastewater treatment by microalgae in different cultivation systems and its importance in biodiesel production. *Fuel* 324:124623. 10.1016/j.fuel.2022.124623

[B13] Attilio C. Alessandro A. C. Erika Y. O. Patrizia P. Marco D. B. (2009). Effect of temperature and nitrogen concentration on the growth and lipid content of Nannochloropsis oculata and Chlorella vulgaris for biodiesel production. *Chem. Eng. Process* 48 1146–1151.

[B14] Aziz M. M. A. Kassim K. A. Shokravi Z. Jakarni F. M. Liu H. Y. Zaini N. (2020). Two-stage cultivation strategy for simultaneous increases in growth rate and lipid content of microalgae: a review. *Renew Sustain. Energy Rev.* 119:109621. 10.1016/j.rser.2019.109621

[B15] Barros A. I. Gonçalves A. L. Simões M. Pires J. C. M. (2015). Harvesting techniques applied to microalgae: a review. *Renew Sustain. Energy Rev.* 41 1489–1500. 10.1016/j.rser.2014.09.037

[B16] Barten R. Djohan Y. Evers W. Wijffels R. Barbosa M. (2021). Towards industrial production of microalgae without temperature control: the effect of diel temperature fluctuations on microalgal physiology. *J. Biotechnol.* 336 56–63. 10.1016/j.jbiotec.2021.06.017 34146615

[B17] Bligh E. G. Dyer W. J. (1959). A rapid method of total lipid extraction and purification. *Can. J. Biochem. Physiol.* 37 911–917. 13671378 10.1139/o59-099

[B18] Breuer G. Lamers P. P. Martens D. E. Draaisma R. B. Wijffels R. H. (2012). The impact of nitrogen starvation on the dynamics of triacylglycerol accumulation in nine microalgae strains. *Bioresour. Technol.* 124 217–226. 10.1016/j.biortech.2012.08.003 22995162

[B19] Cai Q. Song K. Cai P. Tian C. Wang C. Xiao B. (2022). Harvesting of different microalgae through 100-μm-pore-sized screen filtration assisted by cationic polyacrylamide and specific extracellular organic matter. *Sep. Purif. Technol.* 280:119918. 10.1016/j.seppur.2021.119918

[B20] Candice R. E. Sean O. Dorin B. (2019). Central composite design parameterization of microalgae/cyanobacteria co-culture pretreatment for enhanced lipid extraction using an external clamp-on ultrasonic transducer. *Ultrason. Sonochem.* 51 496–503. 10.1016/j.ultsonch.2018.05.006 29793838

[B21] Carullo D. Abera B. D. Casazza A. A. Donsì F. Perego P. Ferrari G. (2018). Effect of pulsed electric fields and high pressure homogenization on the aqueous extraction of intracellular compounds from the microalgae Chlorella vulgaris. *Algal Res.* 31 60–69. 10.1016/j.algal.2018.01.017

[B22] Cheah W. Y. Ling T. C. Show P. L. Juan J. C. Chang J. Lee D. (2016). Cultivation in wastewaters for energy: a microalgae platform. *Appl. Energ.* 179 609–625. 10.1016/j.apenergy.2016.07.015

[B23] Chen K. Wu X. Zou Z. Dong Y. Zhang S. Li X. (2022). Assess heavy metals-induced oxidative stress of microalgae by Electro-Raman combined technique. *Anal. Chim. Acta.* 1208:339791. 10.1016/j.aca.2022.339791 35525583

[B24] Chen Y. M. Liu J. C. Ju Y. (1998). Flotation removal of algae from water. *Colloids Surf. B Biointerfaces.* 12 49–55. 10.1016/S0927-7765(98)00059-9

[B25] Cheng M. Xie X. Schmitz P. Fillaudeau L. (2021). Extensive review about industrial and laboratory dynamic filtration modules: scientific production, configurations and performances. *Sep. Purif. Technol.* 265:118293. 10.1016/j.seppur.2020.118293

[B26] Cheng P. Huang J. Song X. Yao T. Jiang J. Zhou C. (2022). Heterotrophic and mixotrophic cultivation of microalgae to simultaneously achieve furfural wastewater treatment and lipid production. *Bioresour. Technol.* 349:126888. 10.1016/j.biortech.2022.126888 35202828

[B27] Chew K. W. Chia S. R. Show P. L. Yap Y. J. Ling T. C. Chang J. (2018). Effects of water culture medium, cultivation systems and growth modes for microalgae cultivation: a review. *J. Taiwan Inst. Chem. E.* 91 332–344. 10.1016/j.jtice.2018.05.039

[B28] Chhandama M. V. L. Satyan K. B. Changmai B. Vanlalveni C. Rokhum S. L. (2021). Microalgae as a feedstock for the production of biodiesel: a review. *Bioresour. Technol. Rep.* 15:100771. 10.1016/j.biteb.2021.100771

[B29] Choi O. K. Hendren Z. Kim G. D. Dong D. Lee J. W. (2020). Influence of activated sludge derived-extracellular polymeric substance (ASD-EPS) as bio-flocculation of microalgae for biofuel recovery. *Algal Res.* 45:101736. 10.1016/j.algal.2019.101736

[B30] Converti A. Casazza A. A. Ortiz E. Y. Perego P. Del Borghi M. (2009). Effect of temperature and nitrogen concentration on the growth and lipid content of Nannochloropsis oculata and Chlorella vulgaris for biodiesel production. *Chem. Eng. Processing* 48 1146–1151. 10.1016/j.cep.2009.03.006

[B31] Dahlqvist A. Stahl U. Lenman M. Banas A. Lee M. Sandager L. (2000). Phospholipid:diacylglycerol acyltransferase: an enzyme that catalyzes the acyl-CoA-independent formation of triacylglycerol in yeast and plants. *Proc. Natl. Acad. Sci. U S A.* 97 6487–6492. 10.1073/pnas.120067297 10829075 PMC18631

[B32] Di Caprio F. Altimari P. Pagnanelli F. (2018). Effect of Ca2+ concentration on Scenedesmus sp. growth in heterotrophic and photoautotrophic cultivation. *N. Biotechnol.* 40 228–235. 10.1016/j.nbt.2017.09.003 28919374

[B33] Dianursanti, Baharudin T. R. Mohamad T. G. Taufik H. A. (2014). Industrial Tofu Wastewater as a Cultivation Medium of Microalgae Chlorella vulgaris. *Energy Procedia* 47 56–61. 10.1016/j.egypro.2014.01.196

[B34] Dries V. Imogen F. Ilse F. Boudewijn M. Koenraad M. (2011). Flocculation of Chlorella vulgaris induced by high pH: role of magnesium and calcium and practical implications. *Bioresour. Technol.* 105 114–119. 10.1016/j.biortech.2011.11.105 22182473

[B35] Ebhodaghe S. O. Imanah O. E. Ndibe H. (2022). Biofuels from microalgae biomass: a review of conversion processes and procedures. *Arab. J. Chem.* 15:103591. 10.1016/j.arabjc.2021.103591

[B36] Enamala M. K. Enamala S. Chavali M. Donepudi J. Yadavalli R. Kolapalli B. (2018). Production of biofuels from microalgae – A review on cultivation, harvesting, lipid extraction, and numerous applications of microalgae. *Renew Sustain. Energy Rev.* 94 49–68. 10.1016/j.rser.2018.05.012

[B37] Fuad N. Omar R. Kamarudin S. Harun R. Idris A. (2018). Mass harvesting of marine microalgae using different techniques. *Food Bioprod. Process* 112 169–184. 10.1016/j.fbp.2018.10.006

[B38] Gao F. Yang H. Li C. Peng Y. Lu M. Jin W. (2019). Effect of organic carbon to nitrogen ratio in wastewater on growth, nutrient uptake and lipid accumulation of a mixotrophic microalgae Chlorella sp. *Bioresour. Technol.* 282 118–124. 10.1016/j.biortech.2019.03.011 30852331

[B39] Gao P. Guo L. Gao M. Zhao Y. Jin C. She Z. (2022). Regulation of carbon source metabolism in mixotrophic microalgae cultivation in response to light intensity variation. *J. Environ. Manage.* 302:114095. 10.1016/j.jenvman.2021.114095 34775333

[B40] Gerde J. A. Montalbo-Lomboy M. Yao L. Grewell D. Wang T. (2012). Evaluation of microalgae cell disruption by ultrasonic treatment. *Bioresour. Technol.* 125 175–181. 10.1016/j.biortech.2012.08.110 23026331

[B41] Hadiyanto H. Widayat W. Christwardana M. Pratiwi M. E. (2022). The flocculation process of Chlorella sp. using chitosan as a bio-flocculant: optimization of operating conditions by response surface methodology. *Curr. Res. Green Sustain. Chem.* 5:100291. 10.1016/j.crgsc.2022.100291

[B42] He J. Ding W. Han W. Chen Y. Jin W. Zhou X. (2022). A bacterial strain Citrobacter W4 facilitates the bio-flocculation of wastewater cultured microalgae Chlorella pyrenoidosa. *Sci. Total Environ.* 806:151336. 10.1016/j.scitotenv.2021.151336 34743821

[B43] Henderson R. K. Parsons S. A. Jefferson B. (2008a). Surfactants as bubble surface modifiers in the flotation of algae: dissolved air flotation that utilizes a chemically modified bubble surface. *Environ. Sci. Technol.* 42 4883–4888. 10.1021/es702649h 18678021

[B44] Henderson R. K. Parsons S. A. Jefferson B. (2008b). Successful Removal of Algae through the Control of Zeta Potential. *Sep. Sci. Technol.* 43 1653–1666.

[B45] Hongxiang L. Jun C. Yanxia Z. Ke L. Jianglei T. Junhu Z. (2019). Responses of Arthrospira ZJU9000 to high bicarbonate concentration (HCO 3 – : 171.2 mM): how do biomass productivity and lipid content simultaneously increase? *Algal Res.* 41:101531.

[B46] Hou Q. Qi X. Zhen M. Qian H. Nie Y. Bai C. (2021). Biorefinery roadmap based on catalytic production and upgrading 5-hydroxymethylfurfural. *Green Chem.* 23 119–231. 10.1039/d0gc02770g

[B47] Isiramen O. E. Bahri P. A. Moheimani N. R. Vadiveloo A. Shayesteh H. Parlevliet D. A. (2022). Improving pH control and carbon dioxide utilisation efficiency in microalgae cultivation systems with the use of a Proportional-integral + dead-zone control strategy. *Bioresour. Technol. Rep.* 17:100917. 10.1016/j.biteb.2021.100917

[B48] Jafari A. Esmaeilzadeh F. Mowla D. Sadatshojaei E. Heidari S. Wood D. A. (2021). New insights to direct conversion of wet microalgae impregnated with ethanol to biodiesel exploiting extraction with supercritical carbon dioxide. *Fuel* 285:119199. 10.1016/j.fuel.2020.119199

[B49] Japar A. S. Takriff M. S. Mohd Yasin N. H. (2021). Microalgae acclimatization in industrial wastewater and its effect on growth and primary metabolite composition. *Algal Res.* 53:102163. 10.1016/j.algal.2020.102163

[B50] Jarvis P. Buckingham P. Holden B. Jefferson B. (2009). Low energy ballasted flotation. *Water Res.* 43 3427–3434. 10.1016/j.watres.2009.05.003 19524997

[B51] Jon B. S. Lars D. Hans C. E. Terje V. (2019). Temperature dependent growth rate, lipid content and fatty acid composition of the marine cold-water diatom Porosira glacialis. *Algal Res.* 37 11–16.

[B52] Karim A. Islam M. A. Khalid Z. B. Faizal C. K. M. Khan M. M. R. Yousuf A. (2020). “Chapter 9 – Microalgal Cell Disruption and Lipid Extraction Techniques for Potential Biofuel Production,” in *Microalgae Cultivation for Biofuels Production*, ed. Yousuf A. (Cambridge: Academic Press), 129–147.

[B53] Khalid A. A. H. Yaakob Z. Abdullah S. R. S. Takriff M. S. (2019). Assessing the feasibility of microalgae cultivation in agricultural wastewater: the nutrient characteristics. *Environ. Technol. Innov.* 15:100402. 10.1016/j.eti.2019.100402

[B54] Kuluncsics Z. Perdiz D. Brulay E. Muel B. Sage E. (1999). Wavelength dependence of ultraviolet-induced DNA damage distribution: involvement of direct or indirect mechanisms and possible artefacts. *J. Photochem. Photobiol. B: Biol.* 49 71–80. 10.1016/S1011-1344(99)00034-2 10365447

[B55] Laamanen C. A. Ross G. M. Scott J. A. (2016). Flotation harvesting of microalgae. *Renew. Sustain. Energy Rev.* 58 75–86. 10.1016/j.rser.2015.12.293

[B56] Li J. Guo Z. (2017). Structure Evolution of Synthetic Amino Acids-Derived Basic Ionic Liquids for Catalytic Production of Biodiesel. *ACS Sustain. Chem. Eng.* 5 1237–1247. 10.1021/acssuschemeng.6b02732

[B57] Li Y. Kiani H. Tiwari B. K. Halim R. (2022). “11 – Unit operations applied to cell disruption of microalgae,” in *3rd Generation Biofuels*, eds Jacob-Lopes E. Zepka L. Q. Severo I. A. Maroneze M. M. (Sawston, Uk: Woodhead Publishing), 225–248.

[B58] Liu G. Qiao L. Zhang H. Zhao D. Su X. (2014). The effects of illumination factors on the growth and HCO 3 – fixation of microalgae in an experiment culture system. *Energy* 78 40–47. 10.1016/j.energy.2014.05.043

[B59] Liu H. Chen H. Wang S. Liu Q. Li S. Song X. (2018). Optimizing light distribution and controlling biomass concentration by continuously pre-harvesting Spirulina platensis for improving the microalgae production. *Bioresour. Technol.* 252 14–19. 10.1016/j.biortech.2017.12.046 29306124

[B60] Liu J. Tao Y. Wu J. Zhu Y. Gao B. Tang Y. (2014). Effective flocculation of target microalgae with self-flocculating microalgae induced by pH decrease. *Bioresour. Technol.* 167 367–375. 10.1016/j.biortech.2014.06.036 24998477

[B61] Liu S. Rouquié C. Lavenant L. Frappart M. Couallier E. (2022). Coupling bead-milling and microfiltration for the recovery of lipids and proteins from Parachlorella kessleri: impact of the cell disruption conditions on the separation performances. *Sep. Purif. Technol.* 287:120570. 10.1016/j.seppur.2022.120570

[B62] Ma X. Mi Y. Zhao C. Wei Q. (2022). A comprehensive review on carbon source effect of microalgae lipid accumulation for biofuel production. *Sci. Total Environ.* 806:151387. 10.1016/j.scitotenv.2021.151387 34740661

[B63] Maity S. Mallick N. (2022). Trends and advances in sustainable bioethanol production by marine microalgae: a critical review. *J. Clean. Prod.* 345:131153. 10.1016/j.jclepro.2022.131153

[B64] Maria N. M. George P. Ioannis T. K. Nikolaos K. (2019). Effect of Light Intensity and Quality on Growth Rate and Composition of Chlorella vulgaris. *Plants* 9:31. 10.3390/plants9010031 31878279 PMC7020147

[B65] Martin C. de la Noüe J. Picard G. (1985). Intensive cultivation of freshwater microalgae on aerated pig manure. *Biomass* 7 245–259. 10.1016/0144-4565(85)90064-2

[B66] Menegazzo M. L. Fonseca G. G. (2019). Biomass recovery and lipid extraction processes for microalgae biofuels production: a review. *Renew. Sustain. Energy Rev.* 107 87–107. 10.1016/j.rser.2019.01.064

[B67] Min K. H. Kim D. H. Ki M. Pack S. P. (2022). Recent progress in flocculation, dewatering, and drying technologies for microalgae utilization: scalable and low-cost harvesting process development. *Bioresour. Technol.* 344:126404. 10.1016/j.biortech.2021.126404 34826566

[B68] Monika P. R. Trishnamoni G. Nikunj S. (2015). Effect of Salinity, pH, Light Intensity on Growth and Lipid Production of Microalgae for Bioenergy Application. *OnLine J. Biol. Sci.* 15 260–267.

[B69] Moreno-Garcia L. Gariépy Y. Barnabé S. Raghavan G. S. V. (2019). Effect of environmental factors on the biomass and lipid production of microalgae grown in wastewaters. *Algal Res.* 41:101521.

[B70] Mousavi M. Setoodeh P. Farsi M. (2022). Theoretical study of flue gas CO2 conversion to microalgae Chlorella vulgaris biomass in a bubble column photobioreactor: tanks-in-series approach, kinetic modeling, and dynamic optimization. *J. Environ. Chem. Eng.* 10:107868. 10.1016/j.jece.2022.107868

[B71] Munns R. Greenway H. Setter T. L. Kuo J. (1983). Turgor Pressure, Volumetric Elastic Modulus, Osmotic Volume and Ultrastructure of Chlorella emersonii Grown at High and Low External NaCl. *J. Exp. Bot.* 34 144–155. 10.1093/jxb/34.2.144

[B72] Najjar Y. S. H. Abu-Shamleh A. (2020). Harvesting of microalgae by centrifugation for biodiesel production: a review. *Algal Res.* 51:102046. 10.1016/j.algal.2020.102046

[B73] Obeid S. Beaufils N. Camy S. Takache H. Ismail A. Pontalier P. (2018). Supercritical carbon dioxide extraction and fractionation of lipids from freeze-dried microalgae Nannochloropsis oculata and Chlorella vulgaris. *Algal Res.* 34 49–56. 10.1016/j.algal.2018.07.003

[B74] Ortiz A. García J. Uggetti E. Díez-Montero R. (2022). Optimization of multi-stage thickening of biomass in a demonstrative full–scale microalgae-based wastewater treatment system. *Sep. Purif. Technol.* 281:119830. 10.1016/j.seppur.2021.119830

[B75] Ortiz A. García-Galán M. J. García J. Díez-Montero R. (2021). Optimization and operation of a demonstrative full scale microalgae harvesting unit based on coagulation, flocculation and sedimentation. *Sep. Purif. Technol.* 259:118171. 10.1016/j.seppur.2020.118171

[B76] Peng X. Meng F. Wang Y. Yi X. Cui H. (2020). Effect of pH. Temperature, and CO_2 Concentration on Growth and Lipid Accumulation of Nannochloropsis sp. MASCC 11. *J. Ocean U. China.* 19 1183–1192.

[B77] Peperzak L. Colijn F. Koeman R. Gieskes W. W. C. Joordens J. C. A. (2003). Phytoplankton sinking rates in the Rhine region of freshwater influence. *J. Plankton Res.* 25 365–383. 10.1093/plankt/25.4.365

[B78] Phong W. N. Show P. L. Le C. F. Tao Y. Chang J. Ling T. C. (2018). Improving cell disruption efficiency to facilitate protein release from microalgae using chemical and mechanical integrated method. *Biochem. Eng. J.* 135 83–90. 10.1016/j.bej.2018.04.002

[B79] Pittman J. K. Dean A. P. Osundeko O. (2011). The potential of sustainable algal biofuel production using wastewater resources. *Bioresour. Technol.* 102 17–25. 10.1016/j.biortech.2010.06.035 20594826

[B80] Prasanthkumar S. Santhosh K. K. Linu M. Joseph G. R. (2020). Experimental evaluation of the culture parameters for optimum yield of lipids and other nutraceutically valuable compounds in Chloroidium saccharophillum (Kruger) comb. *Nov. Renew. Energ.* 147 1082–1097.

[B81] Purba L. D. A. Othman F. S. Yuzir A. Mohamad S. E. Iwamoto K. Abdullah N. (2022). Enhanced cultivation and lipid production of isolated microalgae strains using municipal wastewater. *Environ. Technol. Innov.* 27:102444. 10.1016/j.eti.2022.102444

[B82] Qi W. Chen T. Wang L. Wu M. Zhao Q. Wei W. (2017). High-strength fermentable wastewater reclamation through a sequential process of anaerobic fermentation followed by microalgae cultivation. *Bioresour. Technol.* 227 317–323. 10.1016/j.biortech.2016.12.062 28040653

[B83] Qian J. Shimotori K. Liu X. Ban S. Akizuki S. Fujiwara M. (2021). Enhancement of algal growth by Mg2+ released from anaerobic digestion effluent of aquatic macrophytes through photolysis. *Biochem. Eng. J.* 172:108065. 10.1016/j.bej.2021.108065

[B84] Ranadheer P. Kona R. Sreeharsha R. V. Venkata Mohan S. (2019). Non-lethal nitrate supplementation enhances photosystem II efficiency in mixotrophic microalgae towards the synthesis of proteins and lipids. *Bioresource Technol.* 283 373–377. 10.1016/j.biortech.2019.03.089 30929825

[B85] Rastogi R. P. Richa, Kumar A. Tyagi M. B. Sinha R. P. (2010). Molecular mechanisms of ultraviolet radiation-induced DNA damage and repair. *J. Nucleic Acids.* 2010:592980. 10.4061/2010/592980 21209706 PMC3010660

[B86] Reddy V. S. Rao D. K. V. Rajasekharan R. (2010). Functional characterization of lysophosphatidic acid phosphatase from Arabidopsis thaliana. *Biochimica et Biophys. Acta (BBA)Mol. Cell Biol. Lipids* 1801 455–461. 10.1016/j.bbalip.2009.12.005 20045079

[B87] Rehman M. Kesharvani S. Dwivedi G. Gidwani Suneja K. (2022). Impact of cultivation conditions on microalgae biomass productivity and lipid content. *Mater. Today Proc.* 56 282–290. 10.1016/j.matpr.2022.01.152

[B88] Ren H. Liu B. Kong F. Zhao L. Xie G. Ren N. (2014). Enhanced lipid accumulation of green microalga Scenedesmus sp. by metal ions and EDTA addition. *Bioresour. Technol.* 169 763–767. 10.1016/j.biortech.2014.06.062 25037828

[B89] Ruiz J. Wijffels R. H. Dominguez M. Barbosa M. J. (2022). Heterotrophic vs autotrophic production of microalgae: bringing some light into the everlasting cost controversy. *Algal Res.* 64:102698. 10.1016/j.algal.2022.102698

[B90] šalić A. Jurinjak Tušek A. Gojun M. Zelić B. (2020). Biodiesel purification in microextractors: choline chloride based deep eutectic solvents vs water. *Sep. Purif. Technol.* 242:116783. 10.1016/j.seppur.2020.116783

[B91] Sanghamitra S. Deshmukh S. Narayan K. P. (2020). Effects of alternate nutrient medium on microalgae biomass and lipid production as a bioenergy source for fuel production. *Mater. Today Proc.* 28 659–664. 10.1016/j.matpr.2019.12.238

[B92] Sanyal D. Venkata Subhash G. Saxena N. Kargupta W. Sapre A. Dasgupta S. (2022). “Chapter 9 – Switchable green solvents for lipids extraction from microalgae,” in *Green Sustainable Process for Chemical and Environmental Engineering and Science*, eds Inamuddin B. R. Asiri A. M. (Amsterdam: Elsevier), 157–176.

[B93] Shankar M. Chhotaray P. K. Agrawal A. Gardas R. L. Tamilarasan K. Rajesh M. (2017). Protic ionic liquid-assisted cell disruption and lipid extraction from fresh water Chlorella and Chlorococcum microalgae. *Algal Res.* 25 228–236. 10.1016/j.algal.2017.05.009

[B94] Sharma K. K. Garg S. Li Y. Malekizadeh A. Schenk P. M. (2014). Critical analysis of current Microalgae dewatering techniques. *Biofuels* 4 397–407. 10.4155/bfs.13.25

[B95] Shelef G. Sukenik A. Green M. (1984). *Microalgae Harvesting and Processing: a Literature Review.* Technical Report ON: TI84013036. Oak Ridge: U.S. Department of Energy Office of Scientific and Technical Information. 10.2172/6204677

[B96] Show K. Lee D. (2014). “Chapter 5 – Algal Biomass Harvesting,” in *Biofuels from Algae*, eds Pandey A. Lee D. Chisti Y. Soccol C. R. (Amsterdam: Elsevier), 85–110. 10.1016/B978-0-444-59558-4.00005-X

[B97] Singh A. Ummalyma S. B. Sahoo D. (2020). Bioremediation and biomass production of microalgae cultivation in river water contaminated with pharmaceutical effluent. *Bioresour. Technol.* 307:123233. 10.1016/j.biortech.2020.123233 32240927

[B98] Sirohi R. Joun J. Lee J. Y. Yu B. S. Sim S. J. (2022). Waste mitigation and resource recovery from food industry wastewater employing microalgae-bacterial consortium. *Bioresour. Technol.* 352:127129. 10.1016/j.biortech.2022.127129 35398537

[B99] Song X. Liu B. Kong F. Ren N. Ren H. (2022). Overview on stress-induced strategies for enhanced microalgae lipid production: application, mechanisms and challenges. *Resour. Conserv. Recycling* 183:106355. 10.1016/j.resconrec.2022.106355

[B100] Sousa C. de Winter L. Janssen M. Vermuë M. H. Wijffels R. H. (2012). Growth of the microalgae Neochloris oleoabundans at high partial oxygen pressures and sub-saturating light intensity. *Bioresour. Technol.* 104 565–570. 10.1016/j.biortech.2011.10.048 22079686

[B101] Soxhlet F. (1879). Die gewichtsanalytische bestimmung des milchfettes. *Dinglers Polytechnisches J.* 232 461–465.

[B102] Spiden E. M. Yap B. H. J. Hill D. R. A. Kentish S. E. Scales P. J. Martin G. J. O. (2013). Quantitative evaluation of the ease of rupture of industrially promising microalgae by high pressure homogenization. *Bioresour. Technol.* 140 165–171. 10.1016/j.biortech.2013.04.074 23688668

[B103] Srinuanpan S. Cheirsilp B. Prasertsan P. Kato Y. Asano Y. (2018). Strategies to increase the potential use of oleaginous microalgae as biodiesel feedstocks: nutrient starvations and cost-effective harvesting process. *Renew. Energ.* 122 507–516. 10.1016/j.renene.2018.01.121

[B104] Su G. Ong H. C. Gan Y. Y. Chen W. Chong C. T. Ok Y. S. (2022). Co-pyrolysis of microalgae and other biomass wastes for the production of high-quality bio-oil: progress and prospective. *Bioresour. Technol.* 344:126096. 10.1016/j.biortech.2021.126096 34626763

[B105] Su Y. (2021). Revisiting carbon, nitrogen, and phosphorus metabolisms in microalgae for wastewater treatment. *Sci. Total Environ.* 762:144590. 10.1016/j.scitotenv.2020.144590 33360454

[B106] Sung Y. J. Lee J. S. Sim S. J. (2022). Accelerated sunlight-driven conversion of industrial flue gas into biofuels by microfluidic high-throughput screening towards improving photosynthesis in microalgae under fluctuating light. *Chem. Eng. J.* 443:136487. 10.1016/j.cej.2022.136487

[B107] Susan M. R. Luong-Van T. George L. David L. P. (2002). Effect of temperature on growth, chemical composition and fatty acid composition of tropical Australian microalgae grown in batch cultures. *Aquaculture* 211 195–214. 10.1016/S0044-8486(01)00875-4

[B108] Sydney T. Marshall-Thompson J. Kapoore R. Vaidyanathan S. Pandhal J. Fairclough J. (2018). The Effect of High-Intensity Ultraviolet Light to Elicit Microalgal Cell Lysis and Enhance Lipid Extraction. *Metabolites.* 8:65. 10.3390/metabo8040065 30326577 PMC6315748

[B109] Taghavijeloudar M. Kebria D. Y. Yaqoubnejad P. (2021). Simultaneous harvesting and extracellular polymeric substances extrusion of microalgae using surfactant: promoting surfactant-assisted flocculation through pH adjustment. *Bioresour. Technol.* 319:124224. 10.1016/j.biortech.2020.124224 33254453

[B110] Teo C. L. Atta M. Bukhari A. Taisir M. Yusuf A. M. Idris A. (2014). Enhancing growth and lipid production of marine microalgae for biodiesel production via the use of different LED wavelengths. *Bioresour. Technol.* 162 38–44. 10.1016/j.biortech.2014.03.113 24736210

[B111] Tran N. T. Seymour J. R. Siboni N. Evenhuis C. R. Tamburic B. (2017). Photosynthetic carbon uptake induces autoflocculation of the marine microalga Nannochloropsis oculata. *Algal Res.* 26 302–311. 10.1016/j.algal.2017.08.005

[B112] Urrutia C. Yañez-Mansilla E. Jeison D. (2019). Bioremoval of heavy metals from metal mine tailings water using microalgae biomass. *Algal Res.* 43:101659. 10.1016/j.algal.2019.101659

[B113] Vignesh P. Pradeep Kumar A. R. Ganesh N. S. Jayaseelan V. Sudhakar K. (2021). Biodiesel and green diesel generation: an overview. *Oil Gas Sci. Technol.* 76:6. 10.2516/ogst/2020088

[B114] Vitova M. Bisova K. Kawano S. Zachleder V. (2015). Accumulation of energy reserves in algae: from cell cycles to biotechnological applications. *Biotechnol. Adv.* 33 1204–1218. 10.1016/j.biotechadv.2015.04.012 25986035

[B115] Wan C. Alam M. A. Zhao X. Zhang X. Guo S. Ho S. (2015). Current progress and future prospect of microalgal biomass harvest using various flocculation technologies. *Bioresour. Technol.* 184 251–257. 10.1016/j.biortech.2014.11.081 25499148

[B116] Wang H. Meng Y. Cao X. Ai J. Zhou J. Xue S. (2015). Coordinated response of photosynthesis, carbon assimilation, and triacylglycerol accumulation to nitrogen starvation in the marine microalgae Isochrysis zhangjiangensis (Haptophyta). *Bioresour. Technol.* 177 282–288. 10.1016/j.biortech.2014.11.028 25496949

[B117] Wang L. Zhang B. (2022). “Chapter 10 – Cultivation of microalgae on agricultural wastewater for recycling energy, water, and fertilizer nutrients,” in *Integrated Wastewater Management and Valorization Using Algal Cultures*, eds Demirer G. N. Uludag-Demirer S. (Amsterdam: Elsevier), 235–264. 10.1016/B978-0-323-85859-5.00006-3

[B118] Wang Q. Shen Q. Wang J. Zhao J. Zhang Z. Lei Z. (2022). Insight into the rapid biogranulation for suspended single-cell microalgae harvesting in wastewater treatment systems: focus on the role of extracellular polymeric substances. *Chem. Eng. J.* 430:132631. 10.1016/j.cej.2021.132631

[B119] Wang S. Wang X. Miao J. Tian Y. (2018). Tofu whey wastewater is a promising basal medium for microalgae culture. *Bioresour. Technol.* 253 79–84. 10.1016/j.biortech.2018.01.012 29331517

[B120] Wiley P. E. Campbell J. E. (2011). Life-Cycle Assessment of Potential Algal Biodiesel Production in the United Kingdom: a Comparison of Raceways and Air-Lift Tubular Bioreactors. *Energy Fuel* 24 4062–4077. 10.1021/ef1003123

[B121] Wu Q. Guo L. Li X. Wang Y. (2021). Effect of phosphorus concentration and light/dark condition on phosphorus uptake and distribution with microalgae. *Bioresour. Technol.* 340:125745. 10.1016/j.biortech.2021.125745 34426241

[B122] Xin L. Hong-Ying H. Yu-Ping Z. (2011). Growth and lipid accumulation properties of a freshwater microalga Scenedesmus sp. under different cultivation temperature. *Bioresour. Technol.* 102 3098–3102. 10.1016/j.biortech.2010.10.055 21055924

[B123] Yan C. Zhu L. Wang Y. (2016). Photosynthetic CO2 uptake by microalgae for biogas upgrading and simultaneously biogas slurry decontamination by using of microalgae photobioreactor under various light wavelengths, light intensities, and photoperiods. *Appl. Energ.* 178 9–18. 10.1016/j.apenergy.2016.06.012

[B124] Yang J. Cao J. Xing G. Yuan H. (2015). Lipid production combined with biosorption and bioaccumulation of cadmium, copper, manganese and zinc by oleaginous microalgae Chlorella minutissima UTEX2341. *Bioresour. Technol.* 175 537–544. 10.1016/j.biortech.2014.10.124 25459865

[B125] Yang Z. Gao F. Liu J. Yang J. Liu M. Ge Y. (2022). Improving sedimentation and lipid production of microalgae in the photobioreactor using saline wastewater. *Bioresour. Technol.* 347:126392. 10.1016/j.biortech.2021.126392 34822986

[B126] Yaping K. Meijing L. Peipei S. Zhaoqi D. Jin L. (2020). High light boosts salinity stress-induced biosynthesis of astaxanthin and lipids in the green alga Chromochloris zofingiensis. *Algal Res.* 50:101976. 10.1016/j.algal.2020.101976

[B127] Zada S. Lu H. Khan S. Iqbal A. Ahmad A. Ahmad A. (2021). Biosorption of iron ions through microalgae from wastewater and soil: optimization and comparative study. *Chemosphere* 265:129172. 10.1016/j.chemosphere.2020.129172 33302204

[B128] Zafar A. M. Javed M. A. Aly Hassan A. Mehmood K. Sahle-Demessie E. (2021). Recent updates on ions and nutrients uptake by halotolerant freshwater and marine microalgae in conditions of high salinity. *J. Water Process Eng.* 44:102382. 10.1016/j.jwpe.2021.102382

[B129] Zeng J. Wang Z. Chen G. (2021). Biological characteristics of energy conversion in carbon fixation by microalgae. *Renew. Sustain. Energy Rev.* 152:111661. 10.1016/j.rser.2021.111661

[B130] Zhang C. Li S. Ho S. (2021). Converting nitrogen and phosphorus wastewater into bioenergy using microalgae-bacteria consortia: a critical review. *Bioresour. Technol.* 342:126056. 10.1016/j.biortech.2021.126056 34601027

[B131] Zhang S. Zhou S. Yang X. Xi W. Zheng K. Chu C. (2020). Effect of operating parameters on hydrothermal liquefaction of corn straw and its life cycle assessment. *Environ. Sci. Pollut. R.* 27 6362–6374. 10.1007/s11356-019-07267-4 31873892

[B132] Zhang X. Wu C. Hu C. Li Y. Sun X. Xu N. (2020). Lipid remodeling associated with chitooligosaccharides-induced heat tolerance of marine macroalgae Gracilariopsis lemaneiformis. *Algal Res.* 52:102113. 10.1016/j.algal.2020.102113

[B133] Zhao G. Wang X. Hong Y. Liu X. Wang Q. Zhai Q. (2022). Attached cultivation of microalgae on rational carriers for swine wastewater treatment and biomass harvesting. *Bioresour. Technol.* 351:127014. 10.1016/j.biortech.2022.127014 35307525

[B134] Zhao Z. Li Y. Muylaert K. Vankelecom I. F. J. (2020). Synergy between membrane filtration and flocculation for harvesting microalgae. *Sep. Purif. Technol.* 240:116603. 10.1016/j.seppur.2020.116603

[B135] Zhong Y. Su Y. Zhang D. She C. Chen N. Chen J. (2022). The spatiotemporal variations in microalgae communities in vertical waters of a subtropical reservoir. *J. Environ. Manage.* 317:115379. 10.1016/j.jenvman.2022.115379 35751236

[B136] Zhou L. Jiang Q. Sun S. Wu Y. Li T. Gao Y. (2022). Acetate stimulates tetracycline biodegradation pathways in bioelectrochemical system. *Sep. Purif. Technol.* 286, 120481. 10.1016/j.seppur.2022.120481

[B137] Zienkiewicz K. Du Z. Ma W. Vollheyde K. Benning C. (2016). Stress-induced neutral lipid biosynthesis in microalgae — Molecular, cellular and physiological insights. *Biochimica et Biophys. Acta (BBA) – Mol. Cell Biol. Lipids* 1861 1269–1281. 10.1016/j.bbalip.2016.02.008 26883557

[B138] Zinkoné T. R. Gifuni I. Lavenant L. Pruvost J. Marchal L. (2018). Bead milling disruption kinetics of microalgae: process modeling, optimization and application to biomolecules recovery from Chlorella sorokiniana. *Bioresour. Technol.* 267 458–465. 10.1016/j.biortech.2018.07.080 30036846

